# Neural representations of the sense of self

**DOI:** 10.2478/v10053-008-0084-2

**Published:** 2011-07-20

**Authors:** William R. Klemm

**Affiliations:** College of Veterinary Medicine & Biomedical Sciences, Texas A&M University, Texas

**Keywords:** consciousness, Avatar, theory of consciousness, nerve impulses, neural networks

## Abstract

The brain constructs representations of what is sensed and thought about in the form of nerve impulses that propagate in circuits and network assemblies (Circuit Impulse Patterns, CIPs). CIP representations of which humans are consciously aware occur in the context of a sense of self. Thus, research on mechanisms of consciousness might benefit from a focus on how a conscious sense of self is represented in brain. Like all senses, the sense of self must be contained in patterns of nerve impulses. Unlike the traditional senses that are registered by impulse flow in relatively simple, pauci-synaptic projection pathways, the sense of self is a system- level phenomenon that may be generated by impulse patterns in widely distributed complex and interacting circuits. The problem for researchers then is to identify the CIPs that are unique to conscious experience. Also likely to be of great relevance to constructing the representation of self are the coherence shifts in activity timing relations among the circuits. Consider that an embodied sense of self is generated and contained as unique combinatorial temporal patterns across multiple neurons in each circuit that contributes to constructing the sense of self. As with other kinds of CIPs, those representing the sense of self can be learned from experience, stored in memory, modified by subsequent experiences, and expressed in the form of decisions, choices, and commands. These CIPs are proposed here to be the actual physical basis for conscious thought and the sense of self. When active in wakefulness or dream states, the CIP representations of self act as an agent of the brain, metaphorically as an avatar. Because the selfhood CIP patterns may only have to represent the self and not directly represent the inner and outer worlds of embodied brain, the self representation should have more degrees of freedom than subconscious mind and may therefore have some capacity for a free-will mind of its own. S everal lines of evidence for this theory are reviewed. Suggested new research includes identifying distinct combinatorially coded impulse patterns and their temporal coherence shifts in defined circuitry, such as neocortical microcolumns. This task might be facilitated by identifying the micro-topography of field-potential oscillatory coherences among various regions and between different frequencies associated with specific conscious mentation. Other approaches can include identifying the changes in discrete conscious operations produced by focal trans-cranial magnetic stimulation.

## INTRODUCTION

Theories for conscious mind range from bizarre to prosaic. Bizarre theories include spiritualistic ideas in which mind is imposed on brain from the outside, as if the brain were some sort of antenna (Nunez, 2010). But even traditional science could yield such theories: a case in point is the theory that quantum mechanics entanglement might influence mind at a distance (Stapp, 2007). Other possible explanations for consciousness might be imagined based on string theory, dark matter, or dark energy if the nature of these new discoveries were understood.

The more popular theories include those based on Bayesian probability (Tolman, 1932; cf. Doya, Ishii, Pouget, & Rao, 2007), or chaos theory (Izhikevich, 2007; Freeman, 2009). These ideas when applied as explanations for consciousness tend to be more metaphor than mechanism. Chaos and Bayesian ideas seem to provide fascinating descriptions, but seem lacking in explanatory power. To understand mind, we may ultimately be forced to invoke mathematical models, subatomic physics, or science that doesn’t exist yet. But we don’t have to invoke some kind of “ghost in the machine” to understand consciousness.

To understand conscious mind we have to understand the other aspects of brain function: non-conscious functions such as spinal and brainstem reflexes and neuroendocrine control. Fortunately, we know a great deal about non-conscious neural machinery that ought to be applicable for explaining conscious mind. Common sense, as well as a great deal of neuroscientific evidence, indicates that the conscious mind emerges from the same place that houses non-conscious and subconscious minds: circuits in the brain.

Theories of consciousness mechanisms have perhaps been hindered by vagueness. A more tangible way to think about consciousness is to regard it as sixth sense, the sense of self. Thus, like all senses the sense of self must have a neural representation based on patterns of nerve impulses. Consider that an embodied sense of self is generated and contained as unique combinatorial patterns across multiple neurons in the same circuit. As with other kinds of Circuit Impulse Patterns (CIPs), those representing the sense of self can be learned from experience, stored in memory, modified by subsequent experiences, and expressed in the form of decisions, choices, and commands. These CIPs could be the actual physical basis for conscious thought and the sense of self.

The question of where to look for sense-of-self CIPs should begin with recognizing the areas of brain that are necessary and sufficient for conscious awareness in the context of self. These areas are well known and constitute what I call the *consciousness system*.

## The Consciousness System

The seminal and well-established work in cats of the 50s by Morruzzi, Magoun, and others (reviewed in Klemm & Vertes, 1990) established that consciousness depends on an “ascending reticular arousal system” (ARAS) in the brainstem that activates the neocortex to generate consciousness. The ARAS receives direct activating collateral input from all traditional senses (except olfaction) and in turn activates the neocortex to produce alert wakefulness. Part of this ascending activating pathway also includes the rostral extension of brainstem reticular neurons that surround the main body of the thalamus. Electrical stimulation of the reticular thalamus evokes the characteristic signs of consciousness, namely, field-potential gamma waves in widespread areas of the neocortex (MacDonald, Fifkova, Jones, & Barth, 1998). Yet another component is the intra-laminar portion of the thalamus, neurons of which have characteristic impulse firing patterns during wakefulness (Steriade & Glenn, 1982). During transitions to wakefulness, intra-laminar thalamic neurons exhibit marked increases in firing, which lag the initial increase in brainstem and basal forebrain cholinergic neurons. Thus, it may be that while brainstem neurons trigger consciousness, intra-laminar thalamic neurons may be needed to sustain it and regulate attention shifts (reviewed by Schiff, 2008). In non-human primates, shifts in attention correlate with field potential oscillations in intra-laminar thalamus of 20-80 Hz (Fries, Reynolds, Rorie, & Desimone, 2001; Murthy & Fetz, 1996; Pesaran, Pezaris, Sahani, Mitra, & Andersen, 2002). Oscillations in this high-frequency range in the neocortex are well-known characteristics of consciousness.

**Figure 1.**
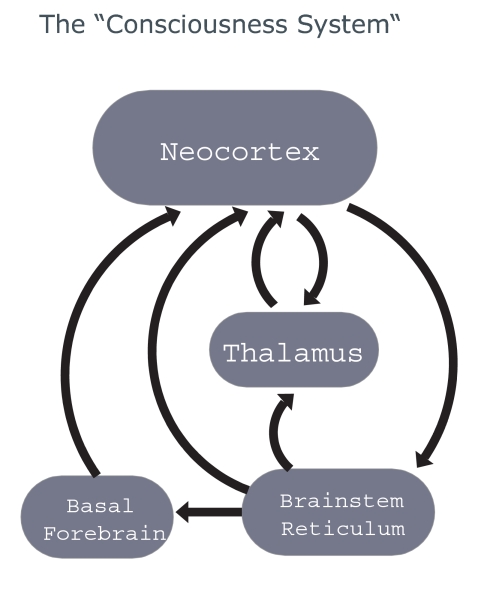
Author’s concept of consciousness as a system function of specific areas of brain.

In addition to the well-known projections from neocortex back into the brainstem reticular area, there are also cortico-fugal projections into the intra-laminar thalamus. Collectively, these interconnected brain areas constitute what could be called a *consciousness system* (Figure 1).

 Steriade and McCarley (2005) vowed to “resurrect” the classic Moruzzi/Magoun studies “from unjustified oblivion.” The often-forgotten consensus is that the ARAS responds to sensory input and creates a cascade of ascending excitatory influences that eventually trigger the cortex into wakefulness and consciousness. The brainstem reticulum integrates converging signals from the viscera, internal milieu, and the bodily senses. It also contains circuitry that regulates vital functions of the heart and respiratory system, sleep and wakefulness cycles, arousal, attention, and the emotions (reviewed by Klemm & Vertes, 1990). Consciousness arises when the outer mantle of brain, the neocortex, is activated (or disinhibited) by influences from the brainstem reticular formation and its rostral extension, the reticular thalamus (Yingling & Skinner, 1977). 

But showing which parts of brain constitute a consciousness system does not explain much. It merely shows where conscious mind comes from and how it might be triggered and sustained. This present review will focus on how consciousness, once triggered, might be produced and sustained.

## Neocortex as the Seat of Consciousness

While the neocortex is only one part of the consciousness system, it is the most crucial part. Focal lesions, as in cardiovascular stroke, for example, produce specific deficiencies in conscious operations. The brainstem and thalamic components of the consciousness system lack the complex network architecture of the neocortex and thus are not likely to do more than limited conscious processing. But the brainstem reticulum is crucial to consciousness, for without the cortical drive it produces, there is permanent coma.

## Cortical Architecture

**Figure 2.**
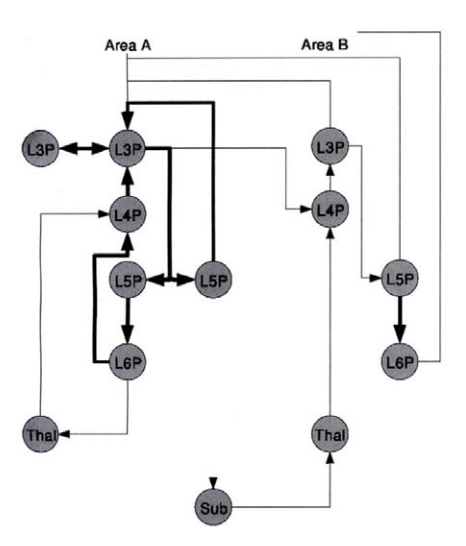
Simplified diagram of the excitatory neurons in any given cortical column (Area A) of the human neocortex and the interconnections with other columns (Area B). The vertical layer location of neurons is indicated by L3, L4, etc. Shown are input sources from subthalamus (Sub) and thalamus (Thal). The nodes of the graph are organized approximately spatially; vertical corresponds to the layers of cortex, and horizontal to its lateral extent. Arrows indicate the direction of excitatory action. Thick edges indicate the relations between excitatory neurons in a local patch of neocortex. Thin edges indicate excitatory connections to and from subcortical structures, and inter-areal connections. Each node is labeled for its cell type. For cortical cells, Lx refers to the layer in which its cell body is located, P indicates that it is an excitatory neuron. Thal = thalamus and Sub = other subcortical structures, such as the basal ganglia. Not shown are the inhibitory neurons and the modulating brainstem inputs, such as noradrenergic neurons in the locus coeruleus, serotonergic neurons in the raphe nuclei, dopaminergic neurons in the ventral tegmental area, and the energizing cholinergic neurons in the nucleus basalis. Top of diagram is the outer surface of cortex, while bottom of diagram shows the deepest layers of cells. Adapted from “Cortical Architecture,” by T. Binzegger, R. J. Douglas, and A. C. Martin, in M. De Gregorio, V. Di Mayo, M. Frucci, & C. Mucio (Eds.), *BVAI 2005. LNCS*, Berlin: Springer-Verlag, p. 21.

Microscopic examination of the neocortex shows that all parts of it have a similar columnar architecture (Douglas & Martin, 2004; see Figure 2). Cortical columns, at their most basic structural level, have their constituent neurons oriented perpendicular to the surface, with its neurons “hard wired” to form a miniature network assembly, typically referred to as a *minicolumn*. Such a small assembly is not likely to have much direct impact on conscious operations, but when columns act in the aggregate much more sophisticated operations become possible. Several adjacent minicolumns form functional aggregates, called *macrocolumns*. About a thousand minicolumns aggregate into a macrocolumn. Macrocolumns have a size of a few millimeters.

 The explanation of columns just given is the common view, but it is simplistic. A recent review by daCosta and Martin (2010) points out that no one has actually seen columns as such. They suggest it is probably more correct and useful to think of cortical columns as “canonical microcircuits.” The idea is that columns are microcircuits repeatedly stacked adjacent to each other, and their intimate cross connections produce the collective emergent functions of cerebral cortex. No one microcircuit stands alone but rather contains only some of the attributes of the whole cortical apparatus. Functions of the canonical microcircuit are dynamic, changing frequently in terms of the subset of neurons that are currently active. 

The general assumption is that column activity oscillates at different frequencies. The important function is the interplay of columns that is governed by shifting degrees of oscillatory synchrony (Freeman, 2007). But let us not lose sight of the fact that the oscillations are caused by CIPs.

Another detail about this circuitry that is not shown in the diagram is that the layer 2 and 3 cells (L2 and L3 cells) get different kinds of input at different levels of their dendrites and cell body. L2 and L3 cells are large pyramidal cells that receive input at different points of their dendritic arborization from three different types of nearby inhibitory cells (reviewed by Jones, 2000). Thus the same cell, and by extension the circuits with which it is associated, can simultaneously contribute to different representations. One representation might be for a specific sensory input, while another might contribute to the representation of the sense of self, thus enabling the conscious sense that it is “I” who sees, hears, and so on.

This idea of impulse patterns as representations is crucial to the thesis herein. Consider how traditional sensory stimuli are registered in the brain. We know from monitoring known anatomical pathways for specific sensations that sensory organs and the brain abstract elements of the outside world and create a representation with CIPs. As long as the CIPs remain active in real time, the sensation is intact, and may even be accessible to consciousness. However, if something disrupts ongoing CIPs to create a different set of CIPs, as for example would happen with a different stimulus, then the original representation disappears and may be lost. Of course, the original CIPs may have been sustained long enough to have been consolidated in memory, in which case retrieval back into active working memory would presumably reconstruct a similar CIP representation of the original stimulus.

We can infer that impulse firing in distributed neocortical circuits is a representation of a perceived stimulus or conscious thought from extension of the classical studies of Hubel and Wiesel (e.g., 1959, 1962). They established that visual images are deconstructed into fragments, with each fragment being represented by impulse discharge of specific neurons. Large numbers of these feature-selective neurons are scattered throughout the visual cortex, each representing its own particular fragmented representation of the over-all image. Such observations have raised the enigma of explaining how all these fragmentary CIP representations are coordinated to reconstruct a conscious percept in the “mind’s eye.” This is now famously referred to as the *binding problem*. Of course, the requirement for binding diverse sensory and cognitive processes extends to numerous brain functions besides vision.

The rich interconnections of various neocortical areas provide a way for the whole complex, once triggered from the brainstem, to operate as one conscious processing system. Note that primary neocortical input comes from the thalamus, terminating in layer 4 (Jones, 1998). Numerous feedback loops are evident. This anatomical substrate for recurrent activity no doubt is a major source of neocortical oscillations of various frequencies (reviewed by Buzsáki, 2006, and Steriade, 2006).

Such organization shows that cortical columns can be mutual regulators. Clusters of adjacent columns can stabilize and become basins of oscillating attraction, and the output to remote regions of cortex can facilitate synchronization with distant basins of attraction (Freeman, 2007). Control in such a system is collective and cooperative.

Elemental cortical circuit design includes recurrent excitatory and inhibitory connections within and between layers (Burkhalter, 2008). Most of the excitatory drive is generated by local recurrent connections within the cortical layers, and the sensory inputs from the outside world are relatively sparse (reviewed by Douglas & Martin, 2004). The usefulness of this design is that weak sensory inputs are amplified by local positive feedback. The risk of such organization is runaway excitation and, in epilepsy, the problem emerges when a lesion removes the normal inhibitory influences that hold the circuitry in check (Jefferys & Whittington, 1996).

Current understanding of neocortical circuitry was discovered in non-human primates. Though human neocortex shows similar anatomical layering and cells types, it is likely that there are some differences in inter-neuronal connectivity. Nonetheless, animal data make clear that neocortex has rich interconnections and capacity for generating multiple oscillatory frequencies with a range of synchronicity possibilities.

The amount of neocortex in humans is relatively much larger than in other primates. But size alone is not sufficient to explain the unique human cognitive abilities and level of consciousness (reviewed by Herculano-Houzel, 2009).

Inhibitory circuits are crucial for controlling oscillations and time-chopping of impulse traffic, both within and among columns (reviewed by Buzsáki, 2006). Some 10-20% of all synapses in neocortex are thought to be inhibitory (reviewed in Douglas & Martin, 2004). We know that neocortex generates multiple-frequency oscillations and that oscillation can time-chop the throughput so that information flows best on every half cycle (reviewed by Buzsáki, 2006, p. 171). Yet no one has identified the temporal succession of impulse patterns for a given mental state, even in a single cortical column. If the impulse activity of multiple neurons in a given column could be recorded simultaneously, then we might have a way to examine the possibilities for combinatorial coding in a given column as it changes with mental state. That may be an insurmountable task, even for a single column, not to mention multiple columns under the same conditions. At a minimum, we could compare a limited set of observations from cortical columns such as undefined multiple-unit activity or field potentials during sleep, anesthesia, alert wakefulness, and dreaming.

## CIP Representations of the Conscious Sense of Self

**Figure 3.**
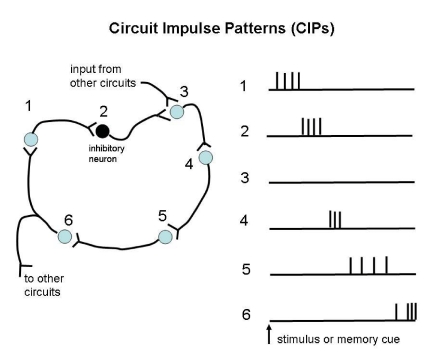
Illustration of the idea of CIPs. In this example small circuit, each neuron generates a certain pattern of spikes, which in turn influences a target neuron. For example, the inhibitory neuron #2 shuts down activity in #3, which nonetheless may reactivate when the inhibition wears off or when excitation comes from another circuit with which it interfaces. Collectively, all the neurons in the circuit constitute a combinatorial CIP for a finite segment of time. When embedded within a network of interfacing circuits, such a CIP may become part of a more global set of combinatorial CIPs that can be regarded as a representation of specific mental states.

The currency of conscious mind is the action potential, or more precisely, the spatial and temporal patterns of impulses in distributed and linked microcircuits and networks (cf. Figure 3). As mentioned, some parts of the so-called *consciousness system* exhibit characteristic firing patterns during alert wakefulness. But all studies of impulse activity at various points along the consciousness system have been performed on neurons without regard to impulse activity in other neurons that are also in the same microcircuit. I wish to emphasize the need to study neurons simultaneously in the same identified circuit, especially in neocortical micro-columns. A given column could perform its functional representations of thought via a combinatorial code across all neurons in the column. This idea of combinatorial coding is central to the theme of this review and will be explored below. While many investigators have reported specific impulse patterns associated with certain conscious functions, what is lacking is identification of combinatorial patterns across multiple neurons in the same circuit.

 Impulses give rise to a wide range of correlates of consciousness (Koch, 2004). But correlates are not always necessary or sufficient to explain consciousness. Not all correlates can be expected to help cause consciousness. Even so, consciousness “presents itself,” as Fingelkurts, Fingelkurts, and Neves (2010a) put it, and Koch would presumably suggest that some sort of neurophysiological processes make that happen. Therefore, looking for correlates seems to have merit as long as the focus is on those correlates that could have a causal effect on conscious sense of self. 

There are those who argue the need to identify a special process within the brain instead of looking for neural correlates. Yet such a process is a correlate. Moreover, that process is generated by neuronal firing. Consciousness is intrinsically experiential and first-person subjective. First-person experiences have to be represented by neuronal activity. So, I extend the sensory representation idea mentioned above to suggest a more global representation of a sense of self, perceived in consciousness.

Fingelkurts, Fingelkurts, and Neves (2010b) say that consciousness “presents itself,” yet is also an emergent property of brain. My interpretation of their explanation is that the operational level of brain organization resides in internal physical brain architecture (i.e., canonical cortical column circuits), and is the basis for conscious sense of self. Thus the operational level ties neurophysiological and subjective domains together. The operational level constitutes consciousness, rather than “emits” it in some mysterious way. Consciousness is self-presenting at the level of operational architectonics of the brain, but is emerging in relation to the neurophysiological level of brain organization.

How is the information of neuronal impulses packaged and distributed? A main purpose of this paper is to encourage neuroscientists to consider the possible usefulness of combinatorial mathematics for the analysis of CIPs. Until now neuroscientists have not found much need to use combinatorial mathematics, which is a well-established math discipline that could be appropriate for testing the role of CIPs in consciousness.

**Figure 4.**
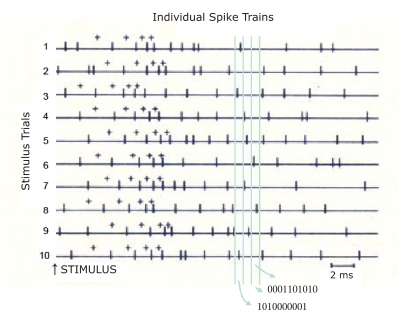
One simple way to identify any existing combinatoric code of spike trains in a defined network. Moving a small time window across simultaneously recorded spike trains allows detection of which neurons produced spikes within that window and the code could be read as a sequence of 0 s and 1 s. In this illustration of 10 simulated spike trains, each has the same conventionally calculated interval histogram. Yet each train contains a “byte” set of serially ordered intervals (expressed here as a “++++” pattern where each interval has a longer duration than the succeeding one). This ordering is not otherwise detectable. At any instant of time (vertical dashed lines) activity within the whole circuit of 10 neurons can be seen to be indexed as a combinatorial coding of impulses; these can even be expressed in quasi-digital form, with presence or absence of an impulse being indicated as a 1 or 0, respectively).

Combinatorial coding, as a principle, clearly operates with certain neural processes, such as taste and odor perception. Also, an argument for combinatorial coding is well established for gene expression in which traits depend on many genes (Kobayashi et al., 2000). We should consider the possibility of application to neural circuit ope-ration. Combinatorial coding could be the “operational level” of consciousness that Fingelkurts and co-workers (2010a, 2010b) espouse. A simple example of how a combinatorial CIP code might be manifest is shown in Figure 4.

Evidence that combinatorial codes exist in cortical circuitry have been detected by Reich et al. in 2001 (Reich, Mechler, & Victor, 2001). They found evidence of combinatorial codes from samples of up to six simultaneously recorded visual cortex neurons. However, they did not confirm that the spike trains came from within the same microcolumn. Recently, Osborne and colleagues (Osborne, Palmer, Lisberger, & Bialek, 2008) report that temporal patterns of spikes and silence across a population of cortical neurons can carry up to twice as much information about visual motion than does population spike count. This result held even when they imposed levels of correlation comparable to those found in cortical recordings. Again, the spike trains were not identified as coming from neurons in the same circuit.

Another neglected area is the sequential ordering of impulses in single spike trains, not to mention ordering across multiple neurons in the same circuit. While historically impulse coding research has focused on firing rate, it is also clear that important information is carried by when impulses occur. Some 30 years ago, there was significant interest in sequential ordering of impulse intervals, but that interest has since waned. One, as yet unfalsified, possibility is that serially ordered intervals could indicate that spike trains are processed as “bytes” (reviewed in Klemm & Sherry, 1982). Although the idea of spike-interval coding has long since been abandoned by most neuroscientists, the evidence for it has not been refuted. If neural circuits do carry some of its information in the form of spike clusters of serially dependent intervals, then mixing input from two or more spike trains could produce an output that preserves the distinct packets of information in ways that could be read and differentiated by other circuits to which it is projected.

This model may be somewhat analogous to the genetic code. While much of DNA is junk (“noise”), there are many isolated unique pieces (“bytes”) that do all the work in highly differentiated ways.

Evidence accumulates that timing of impulses, as opposed to numbers per unit of time, are important to information processing in brain. For example, phase-locking of single units to oscillations seems to be a pre-requisite for successful memory formation (Rutishauser, Ross, Mamelak, & Schuman, 2010). Simulation experiments by Masquelier and colleagues (Masquelier, Hugues, Gustavo, & Thorpe, 2009) recently showed that the phase of impulse firing relative to the oscillation of field potentials provides an important learning scheme. In the last few years, the neuroscience community has shown increasing interest in the role of field-potential coherence in cognitive processes.

Neocortical architecture suggests that its CIP representations either enable conscious awareness or are themselves the essence of consciousness. Of course, CIPs, though essential, are themselves dependent on biochemical processes such as neurotransmitter systems.

## The Created Conscious Sense of Self

Consider the possibility that conscious mind also has its own CIP representation. Specifically, when the brain constructs a sense of self, it must do so via neural representation, which takes the form of unique CIPs. Most neuroscientists might agree that an idea, for example, has a neural representation in a set of CIPs. Is that the same as saying that the CIP is the idea? If the idea can be visualized, then it becomes expressed when the CIPs include those portions of the visual cortex that create the idea in the “mind’s eye.” If the idea can be described verbally, it becomes expressed when the CIPs include the language systems in the left hemisphere.

Just as certain CIPs are a representation of bodily sensations, the brain may also use a unique set of CIPs to generate a consciousness sense of self. When we humans are awake, we are automatically conscious. Given that much of our awake function is performed subconsciously, that means that both states are launched concurrently, from sleep for example. Given that subconscious and conscious functions are so different, each could have different CIP representations, which would in theory be identifiable and distinguishable.

A conscious mind could emerge when subconscious mind achieves a certain “critical mass” of distributed circuit activity that becomes interlinked and coordinated in a unique way. This created conscious mind then becomes available to enrich the processing of subconscious operations. Conscious mind is not aware of the processes of subconscious activity but is aware of the consequences of such activity. No longer is the brain limited to execution of existing programs, but now the introspection of conscious mind allows a deeper consideration of what is being experienced.

Most importantly, the subconscious mind now has another source of programming. Conscious mind provides a new dimension for actively programming the subconscious. In short, conscious mind is the brain’s way of intervening with itself. This goes to the heart of the biological case for free will and personal responsibility. The representations of self may not be devoted to the external and internal worlds of embodied brain, as is required of the representations of subconscious mind. Therefore, the sense of self may be less constrained and may have more degrees of freedom for its operations. In short, a degree of free will may be enabled.

The case for free will is argued elsewhere (Klemm, 2010). Actually, this analysis focused on showing flaws in the research reports many have used to claim free-will is an illusion. Other than anecdote and personal experience, convincing evidence for free will remains to be discovered. For the sake of argument, let us consider the possibility that conscious mind is the “I” of each person, and can sometimes be in control. If one thinks of this as an avatar, the conscious mind avatar not only can control the subconscious but it can also control itself. Conscious mind can choose what to read, what people to associate with, what is good for the individual, what attitudes to hold and adjust, what to believe, and what to do. True, because of pre-existing subconscious programming, some conscious choices are more deterministic than others. But because of conscious mind, everyone can at least become aware of the price being paid for bad choices and have the option to change course, to change brain’s programming accordingly.

It is clear that a brain avatar could make such choices. What is less clear is whether those choices are freely willed. But the neural representation for the sense of self is probably quite different from the representations held in subconscious mind. Subconscious representations are constrained by the realities of the physical world, both inside and outside the body. The conscious avatar has no such constraint, because it’s representations are not necessarily referenced to worldly events. True, the avatar representations are often modified and biased by the output of subconscious programming, as evidence by mental “knee-jerk” responses.

Another area of distinction is the capacity for introspection, which by definition occurs in the consciousness of the avatar. Introspection processes likely have their own neural representations which yield choices, decisions, and commands. Introspection expands the realm of alternatives for what to do. This is equivalent to expanding the degrees of freedom for the avatar’s actions. The avatar could be less rigidly programmed than subconscious mind. Therefore, the information processing occurring in the representations of self should be less deterministic – perhaps to the point of allowing a degree of free will.

I agree with those who say who we are is largely learned through experience Much of this learning has occurred and is “remembered” implicitly and subconsciously (LeDoux, 2002). Consciousness, given the nature of the brain systems that enable it, is able to participate in this learned sense of self. Consistent with this view, LeDoux also contends that the self is constructed. This construction is a life-long learning process, being most evident during childhood. Babies, for example, initially seem to act as if they are an extension of the mother and progressively develop indicators of self-awareness. What they learn about themselves is presumably reflected in their CIPs.

## What CIPs of Consciousness Represent

The brain not only contains CIP representations of things we have experienced, but it also can create CIP representations of things and events that we have never experienced. Creativity is a marvelous mystery. Creating a representation of things never seen nor experienced requires reconstituting in unique ways the CIP representations of things we have seen or experienced. No one knows how the brain decides which circuits to engage to generate creative thought. No one knows why some brains are better at the creative process than others. Nor do we know if brains can be taught to be more creative, or if so, how to do it.

Regardless of what CIPs produce the “I” of consciousness, those processes should also be capable of modifying their processing according to the nature of their output, some of which is represented in the consciousness. When we have a conscious experience, the neural processes that make us aware of the output of those circuits provide a physical substrate for self-adjustment, which may also be manifest in the consciousness. In other words, the brain can control its own consciousness.

If thoughts are tagged in the form of CIPs, how does the brain make itself aware of its own CIPs? Does the brain have some sort of meta-tagging mechanism wherein each CIP is itself tagged in a way so that multiple CIPs, when merged at the same time, now have an emergent property that enables an awareness of what the various CIPs represent? If so, how could any such meta-tagging be accomplished?

The process could operate at both subconscious and conscious levels. The difference for conscious mind, however, could be that conscious mind does not “see” the original stimulus, but mainly “looks in on” the CIP representation being held in subconscious mind (sCIPs). Conscious mind may contain CIP (cCIP) representations of another sort. Namely, the brain creates a separate conscious mind that is representation of self-identity, as opposed to representations of external world. Note the emphasis here is that the self-awareness of consciousness is constructed, rather than emergent. Thus, the sense of self-identity can grow with time, being modified by biological maturation and learning experience, resulting in evolving CIP representations.

One may be tempted to conclude that consciousness is a figment of our imagination. Not so. Our sense of individual identity really does exist, presumably in the form of CIPs. Similar things could be said for subconscious mind. The CIPs are themselves very real and subject to biological forces. They are also subject to what many people would call mentalistic forces, given that those mentalistic forces are actually mediated by CIPs.

This view gives rise to the proposition that conscious mind is a CIP avatar that act as the brain’s active agent, a “free will” partner in brain function that operates in parallel and in conjunction with subconscious mind to make the total brain function more adaptive and powerful than could be achieved with subconscious mind only. Evolutionarily, such co-evolution may be the mechanism that changed pre-humans from zombies to who we are today.

## The Consciousness of Dreaming

The sense of self persists in dreams, and thus we should consider that dreams are a special form of consciousness. In a separate paper, I will present a theory for why humans dream, more specifically, why they have the periodic episodes of rapid-eye movement sleep which promote dreaming. Suffice it to say here that EEG signs during dreaming are similar to those seen in alert wakefulness, and thus the same CIP and coherence mechanisms that operate in causing consciousness may also operate during dreaming.

## Popular Related Views

In the last decade, it has become common for theorists to invoke oscillatory synchronization as the basis for consciousness. The emphasis is usually on electromagnetic fields, which as a practical matter are usually monitored as the EEG or field potentials from within the brain. Cortical column assemblies oscillate because the microcircuits in a mini-column oscillate, and since mini-columns are cross connected, they can couple with each other with varying degrees of time locking. Such functional coupling provides a basis for binding the distributed functions and thus generating unified perceptions and thoughts (Edelman & Tononi, 2000; Singer, 2001).

A flurry of publications in the last few years clearly implicates field potential oscillation and synchrony among brain areas in consciousness. But most researchers have not made the most of their data. For example, one index of degree of consciousness could well be the ratio of gamma activity to activity at other frequencies. Another index could be frequency-band-specific differences in the level and topographic distribution of coherence within and between frequencies.

Not everyone accepts the leading theory that high-frequency coherence mediates the binding of fragmented sensory elements, such as bars and edges in a visual scene, and could similarly bind cognitive processes (Shadlen & Movshon, 1999). Evidence that synchrony promotes binding is indirect and incomplete at best. It may be true, as critics argue, that synchrony is only the signature of sensory (and presumably cognitive) binding. No compelling explanation is yet available for how synchrony actually achieves binding. In any case, changes in synchrony must be generated by underlying combinatorially coded CIPs.

Yet even critics conclude that synchronicity must be important, and it might be uniquely important to the issue of consciousness. To be dismissive of oscillatory synchronization is a kind of physiological nihilism and is not warranted by the huge number of phenomena with which it has been associated (Buzsáki, 2006). Transient synchronization of field potential oscillations reflects the underlying linkage and unified function of large neuronal networks. Consciousness likely depends on large-scale cortical network synchronization in multiple frequency bands (not just the ever-popular 40 Hz). Thus, it may be that it is not binding as such that creates consciousness, but rather the kind of binding or to the accessibility to the product of binding by conscious mind.

For instance a study of EEG coherence responses to ambiguous figure stimuli (Klemm, Li, & Hernandez, 2000), evaluated the cognitive binding associated with the “eureka” of sudden realization of the alternative percept in ambiguous-figure drawings. This cognitive eureka was manifest in widespread spatial coherence in two or more frequency bands. These might even have had meaningful synchronization with each other, but that was not tested. Even so, it is not clear why or how consciousness would arise from multiple-frequency binding unless the coherence in different frequencies carries different information. One frequency might carry the information while another might carry the conscious awareness of the information. Another possibility is that coherence creates consciousness only if enough different areas of the brain share in the coherence. These are compelling questions for future research. Ambiguous-figure stimuli are especially useful because the brain can simultaneously hold a conscious perception and a subconscious representation of the identical physical stimulus of the retina. Also, a human can consciously control which of the alternative percepts are consciously perceived at any given moment.

It seems likely that only a fraction of subconscious processing is accessible at any one time, suggesting that only a sub-set of CIPs could acquire the conditions necessary for consciousness or that access to certain subconscious networks is blocked by inhibition. The corollary is that conscious registration may have limited “carrying capacity,” which is definitely demonstrable in the case of working memory. Maybe this is because the CIPs of consciousness have to hold in awareness and working memory not only the CIP information from the subconscious and ongoing external input but also those for the sense of self and all that it entails.

Oscillation coupling determines which neuronal assemblies communicate at any particular instant, and thus the brain can re-wire itself dynamically on a time scale of milliseconds without any need for changing synaptic hardware (Izhikevich, Desai, Walcott, & Hoppensteadt, 2003). A change in frequency allows various neuronal assemblies to process information with minimal cross interference and even allows neurons or mini-columns to participate in different macro-assemblies simply by changing frequency and coherence coupling.

A recently elaborated theory of consciousness (Fingelkurts et al., 2010a, 2010b) bears some resemblance to the avatar idea in the sense that consciousness is thought to arise as a special form of oscillation and synchronization of field potentials among cortical columns. These voltage fields appear as quasi-stationary epochs (from about 30 ms to 6 s) epochs and reflect the underlying mental operations. Synchronization of many such simple operations produced by transient neuronal assemblies located in different brain areas produce spatial-temporal patterns (operational modules, OM) responsible for complex mental operations. OMs can be further synchronized among each other, forming even more complex OMs. Some OMs are equivalent to thoughts. OMs exist as long as there is synchrony of operations that constitute it. Therefore some OMs “live” longer than others; and, therefore some thoughts are longer than others.

The brain produces a range of long and short thoughts which may operate like “frames” of a motion picture. The frames arise from activity in neurons as they interact in oscillatory fashion within and across their local networks. These frames, separated by interludes of less neuronal participation, are concantenated to produce a stream of thought. Certain kinds of frames give rise to consciousness. Conscious thought arises when the cortical columns creating the frames become sufficiently cross-linked and coordinated.

This view seems to have some limitations. First, the frame idea applies most directly to alpha rhythm which can occur as short epochs of a fixed frequency oscillation where the amplitude waxes and wanes over successive time epochs. However, long sustained epochs of alpha occur in relaxed meditative states, not states of more demanding thinking. Also, a few patently conscious people do not exhibit alpha. There is also the problem that high-frequency oscillations in the gamma range and beyond would seem less likely to exist in segmented frame form and more likely to fuse as a continuum.

The main problem is the underlying question of where the oscillating fields come from. One is led to think the conscious realizations come from the field potentials themselves. Ignored is the underlying role of the CIPs that generate the oscillations in the first place. Oscillatory fields certainly reflect what is happening during thinking, but may not be the cause. They certainly are not equivalent to the underlying discrete CIPs whose flow through circuitry may be modulated by the field-potential environment in which they propagate. However, this point is implicit in the statement in the Fingelkurts’ paper (Fingelkurt et al., 2010a, 2010b) to the effect that thought operations are “indexed” by the EEG. But the key question is: Are field potentials the message or a reflection of the message? Is it the fields that are interacting or the message exchange of combinatorial impulse codes?

## Supporting Evidence for a CIP Theory

There are lines of evidence that support the CIP theory in addition to the rationale just developed. Evidence falls into two categories of predictions:

1. The CIPs, or some manifestation thereof, such as the EEG or field potentials, should change as the state of consciousness changes.

2. Changing the CIPs or their manifestation should change the state of consciousness.

In the first category, the whole history of EEG studies, both in laboratories and in hospital settings, attests to the fact that there is generally a strong correlation between the EEG and the state of consciousness. There are apparent exceptions, but these EEG-behavioral dissociations, as they are called, can be attributed to methodology or to misinterpretations of the state of consciousness (Klemm, 1992).

For example, an apparent arousal EEG does not always suggests consciousness. Reports of EEG in lower animal species, like fish and amphibians, show what seems to be an “activated” EEG even during behavioral quiescence that superficially looks like sleep (Klemm, 1973). What is not clear is the frequency band of such EEGs. Such data were obtained before the age of digital EEG and frequency analysis. We know little about the full frequency band and the coherences of various frequency bands in the EEG of any non-primate species. It is entirely possible that lower animals only have beta activity (less than about 30 per second). Moreover, the degree and topography of coherences have never been subjected to examination in any lower species.

The general well-known observations can be summarized as follows:

1. In the highest state of consciousness and alert wakefulness, the EEG is dominated by low voltage-fast activity, typically including oscillations in the frequency band of 40 and more waves per second.

2. In relaxed, meditative states of consciousness, the EEG is dominated by slower activity, often including so-called alpha waves of 8-12 per second.

3. In emotionally agitated states, the EEG often contains a great deal of so-called theta activity of 4-7 waves per second activity.

4. In drowsy and sleep states, the EEG is dominated by large, irregular slow waves of 1-4 per second.

5. In coma, the trend for slowing of activity continues, but the signal magnitude may be greatly suppressed.

6. In death, there is no EEG signal.

Because the EEG is a manifestation of overall CIP activity and an “envelope” of it, such changes in EEG correlates of consciousness support the notion that it is changes in CIP that create changes in the state of consciousness. Even so, these are just correlations, and correlation is not the same as causation.

More convincing evidence comes when changing the CIPs, either through disease or through some external manipulation, changes the state of consciousness. For instance, massive cerebral strokes may wipe out conscious responsiveness to stimuli from large segments of the body. Injection of a sufficient dose of anesthetic produces immediate change in neural activity and unconsciousness ultimately follows. Naturally occurring epilepsy causes massive, rapid bursts of neural activity that wipe out consciousness. Even during the “auras” that often precede an epileptic attack, there are localized signs of epileptic discharge and the patient is very often consciously aware that a full-blown attack may soon ensue (Schulz et al., 1995).

Another line of evidence comes from the modern experimental technique of transcranial magnetic stimulation. Imposing large magnetic fields across discrete areas of scalp is apparently harmless and produces reversible changes in brain electrical activity that in turn are associated with changes in conscious awareness. A wide range of changes in consciousness functions can be produced depending on the extent of tissue exposed to the magnetic field stimulus (Grafman & Wassermann, 1998; see also Capotosto, Babiloni, Romani, & Corbetta, 2009).

## Unleashing the Self-conscious Avatar

During consciousness, the circuitry of the Avatar learns, memorizes, retrieves, and interprets its representation of self. That construct appears every time the necessary CIP conditions are met, as when we wake up each morning in response to a brainstem reticular formation disinhibition of the cortical circuitry that has kept us asleep.

How can the Avatar get generated each day? There must be a threshold for the non-linear processes that create the conditions for emergence of the Avatar from its memory store. Though some people wake up in the morning more groggy that others, consciousness at least in some people suddenly “comes on,” like a light switch. Although after anesthesia there may be unconscious thrashing about (that’s why they strap you down on the gurney), emergence from anesthesia seems instantaneous. Though we cannot yet specify these processes in detail, we know that once consciousness threshold is reached, the effect must involve CIPs in the consciousness system.

**Figure 5.**
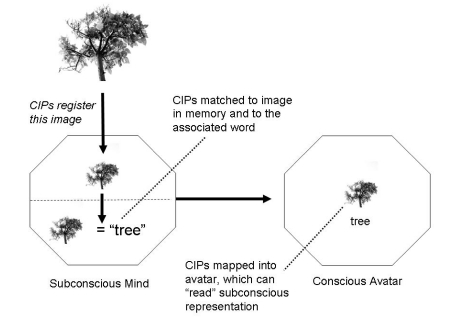
The image is mapped in subconscious mind by a CIP representation. The brain searches its circuits for a template match in memory. When a match occurs, the brain searches further in memory stores for other associations, such as the word *tree* and any emotional associations. The memory CIPs are then accessed by the CIPs of the consciousness Avatar which becomes aware of what the subconscious mind has processed (and does so in its context of self: “I see the tree”).

Could we be consciously aware of our other senses of smell, taste, sight, hearing, etc. without having a sense of self? In the real time during which subconscious mind registers sensations, the consciousness Avatar must also be perceiving the sensations. How can this be? Is there some shared access to sensation? How is the sharing accomplished? Consider the following example in which the eyes detect a tree (Figure 5).

Conscious mind monitors and adjusts as necessary its representation of itself. It also monitors some of the CIP representations of subconscious mind, but presumably has no direct access to the operations of unconscious mind. The representations of self in conscious mind can do other free-will kinds of things, such as reflect on what it knows, plans, decides, and vetoes. In other words, conscious mind is a “mind of its own.”

## Testability of the CIP Avatar Theory

The idea that non-conscious, subconscious, and conscious minds are represented by CIPs seems reasonable. Consciousness research might be better spent trying to falsify CIP hypotheses about mind than with more fanciful ideas such as Bayesian probability, chaos theory, quantum mechanics, dark energy, or others.

Any scientific theory should have the potential for being tested or shown to be false. But how can one possibly test this theory − or any other theory of consciousness? Yet this should not be an excuse to do nothing.

The CIP theory does have the virtue of being based on what we already know to be the currency of information processing in the brain, at least for the non-conscious and subconscious brain. We may not need to invoke metaphors and mathematical models. We do not have to invoke either ghosts or science of the future (such as dark matter or dark energy).

What is it we need experiments to prove? Certainly not the idea that consciousness acts like an Avatar. That is just a metaphor, which has little explanatory power. Metaphors create the illusion of understanding the real thing. Here, the term Avatar is used in an operational way; that is, consciousness is an agent of embodied brain. It is real, not metaphor. We don’t live in some kind of cyberspace like the movie Matrix. We are our consciousness.

Two main experimental approaches seem feasible. One can either disrupt CIPs by external means and monitor resultant changes in conscious thought or attempt to compare CIPs when consciousness is present versus when it is not.

### Disrupting CIPs

We already know that consciousness can be abolished or dramatically disrupted by disruption of CIPs, as with anesthesia, heavy drug sedation, or electroconvulsive shock. A more nuanced approach can be achieved with trans-cranial magnetic stimulation (TCMS; Walsh & Pascual-Leone, 2003). Such stimulation indiscriminately affects both excitatory and inhibitory neurons, but it most certainly disrupts whatever CIPs are present at the time of stimulation. The technique is usually applied focally on specific parts of the neocortex. By selectively altering the CIPs of parts of neocortex that have specific conscious functions, such as language comprehension, musical analysis, or certain conscious spatial tasks, one could demonstrate an association between disruption of CIPs in a given area with disruption of the conscious operations usually performed by that area. Anodal stimulation increases spike firing rates, while cathodal stimulation decreases it.

Some evidence exists that TCMS changes power and phase of EEG oscillation. One study shows that TCMS changed the ratio of alpha to gamma activity over the human parietal cortex, while at the same time increasing the accuracy of a cognitive task (Johnson, Hamidi, & Postle, 2009).

An effect on conscious choice behavior has recently been reported. Anodal stimulation of the dorsolateral prefrontal cortex of the left hemisphere and simultaneous cathodal stimulation of the corresponding area in the right hemisphere changed the freely chosen strategy for guessing whether a random draw from a deck of cards would be red or black, but the opposite stimulus condition did not (Hecht, Walsh, & Lavidor, 2010).

Cognitive responses to TCMS would confirm only a role for CIPs and not the proposed Avatar. But it might be possible by manipulating TCMS pulsing parameters and topography to dissect implicit from explicit operations and show that implicit processing remains while the explicit Avatar function disappears. Use of ambiguous-figure perception could be quite useful here.

It is also important to conduct TCMS studies during sleep and other forms of unconsciousness. Would TCMS of ARAS areas in an awake subject induce sleep? Would TCMS delivered to focal areas of cortex during sleep influence dream content that is specific to that cortical area (such as TCMS of visual cortex inducing visual hallucinations)?

TCMS, however, has its limits. One should expect, for example, that applying focal TCMS over the part of cortex that recognizes specific objects would alter the subject’s cognitive responses. But such studies might yield interesting findings about whether the subject knows errors are being made if corrective feedback is not supplied.

### Monitoring CIPs

We can never describe the CIPs of consciousness until we can record what they are. That would require simultaneous recording of impulse patterns from many neurons in identified circuits. Such a process is expedited by knowing in advance which brain areas are necessary for generating a given conscious operation. The topography of specific conscious operations could be identified with field-potential coherences and/or TCMS.

To monitor CIPs successfully, we may need better methods of impulse pattern detection and description. Combinatorial mathematics will likely be a necessary tool in such investigations. We will also need better methods for examining shifting patterns of synchrony of multiple units or field potentials among multiple impulse generators.

To detect CIPs most meaningfully, investigators may need to identify combinatorial patterns of nerve impulses at successive time increments, but also look for embedded serially ordered impulse interval “bytes” across each neuron in the circuit. For example, if a “+++−“ pattern occurs non-randomly in one neuron during a given cognitive state, there may be temporal linkage to that or some other ordered pattern elsewhere in the circuit.

To monitor impulse activity in distributed circuits could require hundreds, even thousands of microelectrodes implanted directly into the brain. A more limited approach would be akin to that mentioned above, wherein one characterizes CIPs in a limited section (microcolumn) of cortex that is associated with specific conscious functions. It may suffice just to monitor a few of the neurons in a given identified circuit. Good candidate conscious functions might include touch perception, language comprehension, musical analysis, or certain conscious spatial tasks. Perhaps an optical method can be developed where impulse-sensitive dyes can display, in three dimensions, the impulse activity coming from individual neurons. Also, to the extent that coherence may be a key mechanism, we need more robust statistical methods that get beyond pair-wise correlation coefficients to detect coherence of activity from multiple locations.

Animal studies provide the best chance to place multiple-electrode arrays into multiple cortical columns and thereby observe associations of certain CIPs with certain cognitive processes, ranging from simple choice behavior to learning-to-learn situations. TCMS application to these areas could help determine if the CIPs are mere correlates or causally involved. Nanotechnology may lead the way in providing the needed electrode arrays. At a minimum, such electrodes would have to be placed in one cortical column, one or more adjacent columns, and one or more remote columns that is known to have hard-wired connection. There is the problem of course of what we assume about animal consciousness, a problem that diminishes the higher up the phylogenetic scale one goes. Studies in humans, such as patients with severe epilepsy that require electrode placement, might be feasible. Studies of this kind could also shed some light on the role of combinatorial coding for conscious processes.

It is entirely possible that if combinatorially coded CIP changes cause consciousness, they would be expressed as changed field-potential patterns in topographical and co-frequency coherence. Several important comparisons should be made. We can, for example, compare normal adults with babies at various stages of their brain’s maturation. Another way to test is to compare activity in comatose patients (locked-in state, and persistent vegetative state) with normal subjects.

An alternative to recording from multiple units is to let averaged evoked response potentials (ERP) in multiple cortical locations serve as the index of differential CIPs. The usefulness of ERP for monitoring conscious operations has been documented in such studies as early language learning in babies. ERP signatures of phonetic learning are evident at 11 months, responses to known words at 14 months, and syntactic and semantic learning at 2.5 years (Kuhl & Rivera-Gaxiola, 2008). ERP approaches could allow us to monitor impulse activity at a population level and identify evoked responses in different cortical areas under conditions when conscious awareness is manipulated, as with sleep or drugs. Changing conscious state would surely produce topographical changes in evoked response, which in turn can only be caused by changes in CIPs. This would not prove the existence of the Avatar, but it could certainly prove a role for CIPs in consciousness.

Finally, quantitative EEG signals from numerous locations might indirectly indicate meaningful evidence of the elusive Avatar. Special attention should be paid to the topography of coherence patterns and coherences among frequencies. Comparison of such parameters in different states, such as sleep and wakefulness, or under different TCMS conditions, would be essential. Also helpful would be comparison of EEG coherences in babies as their own sense of self develops. Studies could be tied to the age at which self-recognition in a mirror emerges.

## Issues in Test Design

The CIPs and frequency coherences must surely differ between subconscious thinking and conscious thinking. This poses significant problems, since both processes presumably operate in parallel at roughly the same time. With these basic assumptions, a few questions arise that could influence design of experiments:

1. How can we identify specific conscious functions and their associated neural activity?

For example, we can design experiments that will record from or manipulate specific cortical areas known to mediate specific conscious functions, such as speech centers, somatosensory cortex, premotor neocortex, and mirror-neuron zones.

More studies are needed with ambiguous figures. The beauty of evaluating perception of ambiguous figures is that one can compare the same image when it is consciously perceived and when it is not. Evaluating combinatorially coded CIPs from defined circuits in humans may not be feasible (electrodes implanted to detect epileptic foci are not normally placed in the areas of neocortex that would be most useful for study of visual percepts). CIPs might be amenable to study in monkeys, assuming some clever artist can design ambiguous figures that have biological meaning to monkeys (such as a drawing that could be interpreted either as an apple or as a pear). Certainly coherence studies such as the one my lab performed can be extended in humans, and focal TCMS can be used to see how the percept can be changed.

2. How can we distinguish subconscious and conscious thinking under otherwise comparable conditions?

Perhaps this might be accomplished by comparing a classically conditioned response (subconscious) with the same motor activity generated through conscious and voluntary decision.

3. Is the distinction between subconscious and conscious processes attributable to CIPs or to frequency coherences or both?

Obviously, the experiment ideally would examine both combinatorial coding of CIPs and frequency coherences of field potentials recorded at the same time and under the same conditions.

4. Are the distinguishing characteristics of subconscious and conscious thinking restricted to the specific cortical area under investigation or do other more distant brain areas differentially participate, depending on whether the thought is subconscious or conscious?

Obviously, the design should also include monitoring of other cortical areas that directly connect to the specific conscious processing areas.

5. What kinds of discrete conscious thoughts might be useful?

Possible tasks could include word priming (speech centers), willful intent to make certain movements (premotor cortex), or situations where an observer witnesses an action by another that takes place within and without the observer’s personal space (mirror neuron sites). The latter approach can be tested in monkeys, where distinct mirror neurons can be identified, or in humans where fMRI methods can identify areas which appear to function as a “mirror neuron system” (Iacoboni et al., 1999).

6. Can we know if the CIPs and frequency coherences of subconscious thinking occupy the same circuitry as do those of conscious thinking? Is the neural activity synchronous during both kinds of thinking or is there a phase lag?

It would seem necessary to simultaneously monitor neural activity in several places, such as adjacent cortical columns and columns in the other hemisphere that are directly connected.

7. How can we distinguish between the “noise” of background neural activity of consciousness as a global state of special awareness and the activity associated with specific conscious thought?

Experiments must include a conscious null-state in which daydreaming is minimized, and perhaps avoided altogether by including some kind of conscious focus on a single task concurrent with the conscious thought task under investigation. For example, one might require a subject to operate a joy stick that tracks a slowly moving target on a computer screen while at the same time performing the conscious task under investigation.

8. What possible neural mechanisms could provide the “Avatar” circuitry with a “free-will agency” capability that is not found in subconscious mind?

Experiments should compare a subject’s performance of the conscious task at freely selected intervals, rather than on cue. Alternatively, the subject could function in a cued mode, but freely choose whether to generate or withhold response to the cue. The experiments can be based on electrical recordings of previously discovered CIP or frequency coherence signatures of a specific conscious thought or when subjects attempt the task when the cortical areas are temporarily disabled, was with local anesthetic or focal TCMS.

## Conclusions

Brains construct representations of what they detect and think about. The representations take the form of patterned nerve impulses propagating through circuits and networks (circuit impulse patterns, CIPs). This representational scheme has been unequivocally demonstrated for both non-conscious and subconscious minds.

Conscious mind must also be a CIP representation, but unique in that the constructed representations are of a sixth sense of self, an awareness of embodied self and what the self encounters and engages. Thus, this mind may automatically know what it is knowing. This representation is actually an agent, more or less equivalent to an Avatar, serving the brain’s interests and imperatives. The conscious Avatar knows information the same way the non-conscious mind does; that is, through CIP representations of that information. So, the key question is “What is different about the CIPs of consciousness and those of non-consciousness or sub-consciousness?” The CIPs of the Avatar likely differ in spatial and temporal distribution.

The Avatar is a CIP representation itself but also an interpreter of the representations in the brain. It interprets not just linguistically, but also in such terms as non-verbalized sensations, reinforcement contingencies, emotions, and probable outcomes of action alternatives. The Avatar probably has more degrees of operational freedom and could act as a “free will” partner that operates in parallel and in conjunction with subconscious mind to make the total brain function more adaptive and powerful than could be achieved with subconscious mind only (Klemm, 2010).

CIPs, as the currency of thought, seem essential for consciousness. Still, the CIP hypothesis may not be sufficient to understand conscious mind. But research on the CIPs and associated field potentials associated with consciousness seems at least as justifiable as research on the other theories of Bayesian probability, chaos theory, and quantum mechanics. Conscious mind may operate in many ways like everything else the brain does. We don’t necessarily have to invoke mathematical models or particle physics or dark matter/dark energy. Conscious mind constructs CIP representations just as do subconscious and non-conscious minds, with the difference that what is represented in conscious mind is not the outside world or the world of the body, but rather the world of ego. Conscious mind is a CIP representation of the sense of self. This identity is learned, beginning with the fetus and newborn, and develops as the brain develops capacity to represent itself consciously. In short, you have learned to be you. I have learned to be me. Our Avatar nature enables us to change who we are.

The brain creates a CIP representation of its embodied self using visual, tactile, and proprioceptive sensations. Added to this is a representation of personal space that includes a representation of the self in three dimensional space. Long-term memory stores this representation and it is released for operation and updating whenever consciousness is triggered.

The Avatar CIPs are accessible to the subconscious mind operations that generate the Avatar. The brain knows that it has this Avatar and knows what it is doing. Stimuli and assorted thoughts are not isolated. The Avatar knows consciously because its information is processed within the Avatar’s CIP representation of the sense of self. This representation is the awareness and the attendant thought.

Proposed here is the idea that conscious perception arises from combinatorial coding of CIPs. We don’t really know what combinatorial coding means, other than to make the less-than-helpful conclusion that the whole is greater than the sum of its parts. At least, however, we have good reason to believe that what is being coded is the spatio-temporal distribution of spikes and the field potentials they generate in multiple linked neurons.

The needed research tools are at hand for identifying the neural causes of the conscious sense of self. Let the race begin.
